# Improving the bioremediation technology of contaminated wastewater using biosurfactants produced by novel bacillus isolates

**DOI:** 10.1016/j.heliyon.2021.e08616

**Published:** 2021-12-17

**Authors:** Osama M. Darwesh, Mohamed S. Mahmoud, Kholoud M. Barakat, Ayman Abuellil, Maged E. Ahmad

**Affiliations:** a Agricultural Microbiology Department, National Research Centre, Dokki, Cairo, 12622, Egypt; b Botany and Microbiology Department, Faculty of Science, Beni Suief University, Beni Suief, Egypt; c National Institute of Oceanography & Fisheries (NIOF), Egypt

**Keywords:** Biosurfactant, Bioremediation, *Bacillus thuringiensis*, *Bacillus toyonensis*, Antimicrobial activity, Petroleum oil residues

## Abstract

Biosurfactants have many advantages outside chemical one, led for application it through different sectors. So, the present study aimed for improving the bioremediation technology of contaminated wastewater using biosurfactants produced by novel bacillus isolates. In this regard, *Bacillus thuringiensis* and *Bacillus toyonensis* strains were obtained as most producing isolates of highly active biosurfactants. The optimized conditions for high biosurfactants yield production were established. Also, the stability of the produced biosurfactants at various conditions, pH, temperature and salinity was studied. The biosurfactant has been reported up to 120 °C, pH 12 and 10% of NaCl. The identified biosurfactants, decanoic acid and oleamide were applied for wastewater remediation from oil residues and pathogens contamination. The biosurfactant was had high antibacterial activity compared with references antimicrobial drugs, as well as it is enhanced bioremediation technology for petroleum oil residues contaminating sites. Thus, we can say, these biosurfactants could achieve the objectives of sustainable development.

## 1 Introduction

Biosurfactants have a great potential due to their advantages over synthetic one especially in the sectors of environment (Ashraf et al., 2017; Barakat et al., 2017), food industry (Nitschke and Silva, 2018), agriculture (Sachdev and Cameotra, 2013), biomedical products (Singh and Cameotra, 2004; Saha et al., 2018) and nanotechnology (Singh et al., 2017). Because of their environmental compatibility, higher biodegradability, structural diversity and lower critical micelle concentration (CMC), the biosurfactant agents have attracted much attention and led to enhance oil recovery (Geetha et al., 2018), pesticide formulations, ceramic processing, pulp and paper, coal, uranium ore-processing, textiles, detergents and health care (Shoeb et al., 2013; Zhang et al., 2021). Biosurfactants advantages over chemical surfactants by some properties like low toxicity, thermal stability, pH stability and saline resistance (Brasileiro et al., 2015; Varjani and Upasani, 2017). Unfortunately, their industrial production and widely application are presently limited due to high production cost when compared with synthetic surfactant (Sivapathasekaran et al., 2010; Kiran et al., 2010). Hence, biosurfactants have noted as microbial products by high values, that have become an important product for biotechnology application in medical and industry (Nitschke and costa, 2007; Makkar et al., 2011). It may be produced inside the cells (intracellular) or as extracellular agent (Antoniou et al., 2015).

Biosurfactants as surface-active compounds with low molecular weight are commonly manufactured by bacteria (the major biosurfactant producers among others), fungi and yeast. Bacterial strains produce various types of biosurfactants. Species of *Bacillus*, *Pseudomonas*, *Rhodococcus* and *Candida* are noted as the most widely producers of different biosurfactants type under various growth conditions as water-immiscible substrates (Chrzanowski et al., 2012; Oliveira et al., 2015; Luna et al., 2016; Barakat et al., 2017). Based on biosurfactants origin and chemical nature, it can be grouped as lipoproteins, lipopeptides, phospholipids, glycolipids, polymeric molecules and fatty acids (Varjani and Upasani, 2017). Also, it is grouped as low molecular weight (lipopeptides, rhamnolipids, trehalolipids and sophorolipids) and high molecular weight (polymeric molecules and lipoprotein) based on the molecular weight (Uzoigwe et al., 2015). The biosurfactants can apply for enhancing the bioremediation processes by solubilization, emulsification and mobilization actions. The microbial surfactant caused improving solubility of hydrophobic organic compounds like diesel for effective bio-augmentation. However, the degradation process dependents on presence especial species of microbes, water, inorganic nutrients, composition of hydrocarbon, pH, temperature and aeration. Pradeep and his co-authors (2012) bioremediated oil contaminated soil using 4 g (biosurfactant produced by *Pseudomonas aeruginosa*) per kg soil.

Many biosurfactants have exposed activity against pathogenic bacteria, fungi, viruses and algae (Muthusamy et al., 2008). The high require for novel antimicrobial compounds with increasing resistance property of pathogenic microbes against current drugs has attention to apply biosurfactants as antimicrobial compounds (Bĕhal, 2006; Darwesh et al., 2019a). Some biosurfactants stated to be appropriate replacements to antimicrobial materials and synthetic medicines and may be applied as effective and safe therapeutic materials (Cameotra and Makkar, 2004; Singh and Cameotra, 2004; Banat et al., 2010). They show a wide range of antimicrobial goods and hereafter are subjugated for cosmetic, biomedical activities, pharmaceutical and food applications (Uzoigwe et al., 2015; Elshikh et al., 2016).

Depending on the head group nature, surfactants commonly classified into four categories, anionic, cationic, nonionic and amphoteric (Kim et al., 2002; Baltz et al., 2005; Saini et al., 2008). The anionic or cationic hydrophilic head is binding with water due to the presence of negative or positive charge on it (Abalos et al., 2001; Benincasa et al., 2004). The production of biosurfactant returned to the producer strain, culture conditions, the nitrogen and carbon source, C/N ratio, nutritional limits, chemical and physical parameters as pH, temperature, aeration and salinity (Makkar et al., 2011; Mouafi et al., 2016). So, the present work aims to bioremediate the oil contaminated wastewater using biosurfactant compounds produced by novel *Bacillus* strains.

## 2 Materials and methods

### 2.1 Collection of oil contaminated soil and water samples

The samples used for the isolation of bacteria able to produce biosurfactants were collected from oil-contaminated places. Approximate 500 g samples of soil were collected into sterilized bags from the subsurface layer (5–20 cm in depth) to get microbes which didn't affect by sun UV light. Also, about 500 ml water or wastewater samples were collected from contaminated sites by oil residues. The samples were labeled before being transferred to the lab under aseptic conditions. The details of sampling areas and samples properties were represented in Fig. (S1) and Table (S1).

### 2.2 Isolation and purification of biosurfactant producing bacteria

Soil sample (approximate 10 g) was mixed with sterile tap water (100 mL) and shacked for 1 h, and then left another half hour to settle the soil particles. Ten mL of the unsettled solution or water and wastewater samples were added to broth mineral salt medium (MSM) (100 mL) contained 1 % (v/v) motor oil as a carbon source (Yateem et al., 2002)*.* The MSM composition was (g/L): NH_4_NO_3_, 3.00; K_2_HPO_4_, 0.50; KH_2_PO_4_, 1.14; FeSO_4_.7H_2_O, 0.04; NaCl, 0.10; MgSO_4_.7H_2_O, 0.2; CaCl_2_. Enrichment process was done at 35 °C and 120 rpm of shaking in orbital shaker and incubated for three weeks. Then, the enrichment culture was repeated by transferring 10 mL of old culture into another flask containing 90 mL of fresh MSM with motor oil and incubated for another one week (Darwesh et al., 2020a). Isolation of biosurfactant producing bacteria was done by spreading and streaking plate technique (Darwesh et al., 2018). The bacterial cultures (100 μL) were inoculated by streaking onto MSM agar containing 1 % motor oil as a sole carbon source with pH 7 and incubated for 7 days at 35 °C. The morphologically distinctive bacterial colonies were transferred to fresh agar plates for purification and then stored onto agar slants for further screening. The pure culture were slanted and preserved in refrigerator and sub-cultured every 2 months.

### 2.3 Screening of the isolated bacteria for biosurfactant production

The ability of bacterial isolates for biosurfactant agent's production was assessed by numerous screening techniques including oil spreading technique (OST), emulsification index (E_24_) (Barakat et al., 2017), hemolytic assay (HA) (Astuti et al., 2019), cetyl tri methyl ammonium bromide (CTAB) test (Pinzon and Ju, 2009) and surface tension (ST) assay method (Al-Sulaimani et al., 2011).

### 2.4 Identification of the most active biosurfactant-producing bacteria

Two isolates were noted as the most active bacteria at screening step for biosurfactant production. The morphology of these isolates was recorded using light microscopy after gram staining. Then the biochemical testes were done according to Bergey's Manual of Systematic Bacteriology. Also, molecular identification was done on the obtained bacteria to confirm identification tools as described by Darwesh et al. (2014 & 2019b). The genetic material (DNA) was isolated, purified and the 16s rDNA gene was amplified using the reported primers (Kheiralla et al., 2016). The primers used for amplification were 16RW01 (5-AACTGGAGGAAGGTGGGAT-3) as forward primer and 16DG74 (5-AGGAGGTGATCCAACCGCA-3) as reverse one.

### 2.5 Optimization of the biosurfactant production using Plackett-Burman experimental design

The limitations influencing the biosurfactant production were screened using Plackett-Burman design and the experiments were done in triplicates by PBD (Plackett and Burman, 1946; Korayem et al., 2015). A set of nine experiments constructed for seven factors (Table S2) including both medium components and culture conditions: (1) NH_4_NO_3_, (2) KH_2_PO_4,_ (3) K_2_HPO_4_, (4) yeast extract, (5) temperature, (6) pH and (7) inoculum size. The main effect of each factor was determined using Microsoft excel and statistical t-values for the two samples which calculated to determine the significant variable(s) (Darwesh et al., 2020b).

### 2.6 Effect of incubation period on the biosurfactant production

The broth medium (100 mL) was inoculated by bacterial strains and incubated at 30 °C for different incubation periods from zero to 14 days. The samples were collected every 2 days and tested for both oil displacement and E_24_ % evaluation tests. Optical density was measured at 550 nm for one mL culture broth.

### 2.7 Determination of biosurfactant

The cell free filtrate (CFF) (1 mL) was vortexed for 30 s with 0.003 % methylene blue (1 mL), and then mixed with chloroform (an equal volume) for 20 min. The blue surfactant migrated into chloroform layer was collected by centrifugation (3000 rpm for 5 min). The extracted biosurfactant mixture was measured at 625 nm in contradiction of chloroform (pure grade). The standard biosurfactant curve was performed using SDS and then the produced biosurfactant concentrations (g/L) were calculated.

### 2.8 Biosurfactant production, extraction and purification

In this part, the most potent bacterial strains were grown into optimized production medium. The bacterial biomass was eradicated by centrifugation under cooling conditions (6000 rpm for 20 min). The extraction technology (combination of acid precipitation and solvent extraction) was performed according to Vater et al. (2002). In order for precipitation of biosurfactant (lipids and proteins), 6M HCl solution was added to the supernatant to bring final pH of 2.0 and kept the mixture at 4 °C for 12 h. All precipitates were harvested by centrifugation (8000 rpm for 20 min), and extracted 3 times by chloroform/methanol (2:1 v/v). The organic phase was collected and evaporated left an oil-like appearance as a crude biosurfactant (Liu et al., 2011; Joshi and Desai, 2013).

### 2.9 Characterization of the active purified compound using gas chromatography–mass spectrometry (GC-MS)

The GC-MS analysis using Agilent, GC-MS instrument was performed at the National Institute of Oceanography and Fisheries (NIOF), Alexandria, Egypt. The GC-MS conditions were adjusted as detailed by Monteiro et al. (2007). The produced components were identified by comparing their retention time with authentic samples and mass spectra with those of Wiley 275 Library (Wiley, 2006).

### 2.10 Fourier transform infrared spectroscopy (FTIR) characterization of biosurfactant

FTIR investigation was approved to discover the presence of functional groups in the produced biosurfactant and partial identification of these groups (Bagewadi et al., 2018). A 1 mg of the sample was mixed with potassium bromide and pressed (30 s) until appear the translucent pellets. The IR spectra were scanned from 400 to 4000 cm^−1^ using Perkin Elmer grating 100 IR (Norwalk, CT, USA).

### 2.11 Evaluation of the produced biosurfactant stability

The produced biosurfactant agent stability was carried out as previously described (Obayori et al., 2009; Mouafi et al., 2016) against pH, temperature and salt (NaCl) stress. To examine the thermal stability of the produced biosurfactant, the purified compound was heated at 50, 60, 70, 80, 100 and 120 °C for 15 min using water bath and cooled in room temperature, the E_24_ and oil displacement activity was determined. In case of pH stability, 6 pH values of the biosurfactant (acidic; 2, 4 and 6; alkaline 8, 10 and 12) was adjusted using HCl or NaOH. The effect of NaCl concentrations on biosurfactant stability was measured at 2, 4, 6, 8 and 10 %.

### 2.12 Bioremediation of contaminated wastewater using the produced biosurfactants

#### 2.12.1 Assay of antimicrobial activity

The antibacterial activity of the produced biosurfactant was evaluated using disk diffusion method on nutrient agar medium (Marrez et al., 2019; Mourad et al., 2020). Some reference pathogenic bacteria were applied in this experiment, they like gram-positive bacteria (*Bacillus cereus*, *Staphylococcus aureus* and *Streptococcus pyogenes*) and gram-negative bacteria (*Klebseilla pneumonia*, *Pseudomonas aeruginosa* and *Acenetobacter sp.*). Disk of 6.0 mm in diameter was saturated with proper volume of the produced biosurfactant until dryness, and then the disk was applied on the surface of agar medium seeded with target organisms. The sensitivity of the microorganism species to the tested substances was determined by measuring the size of inhibitory growth (clear zone) around the disks. The size of clear zone is proportional to the inhibitory action of the compound under investigation. Five formulations of antibiotic-loaded discs such as Amikacin, Amoxicillin/clavulanic, Ciprofloxacin, Piperacillin and Chloramphenicol were used as standard references antibiotics. Moreover, the capacity for inhibition the pathogens was measured for comparing with the bioactivity of purified biosurfactant (Ali et al., 2016; Sultan et al., 2016).

#### 2.12.2 Bioremediation and/or degradation of oil contaminated wastewater

In this section of work, the wastewater samples containing oil residues were used without sterilization to study the behavior of the obtained bacterial strains with the indigenous microflora that presents normally in the collected samples. The control samples were the wastewater samples as it is, without adding any nutrient or bacteria and the treated flasks were inoculated by bacterial strains. The flasks were incubated under shaking conditions (120 rpm) at 30 °C. After 14 h of incubation, the oil degradation was detected. On the other hand, the same steps were repeated but with adding nutrients (medium ingredient) to wastewater samples.

## 3 Results and discussion

### 3.1 Isolation and screening of biosurfactant producing bacteria

Biosurfactant and bioemulsifier agents are very suitable alternatives to chemical surfactants due to their useful properties like biodegradability, eco-friendly, bioavailability, less/no toxicity, high specificity, selectivity at temperature, pH, salinity and may synthesis from cheaper renewable substrates (Satpute et al., 2010). For that, fourteen samples were collected from oil-contaminated habitats: soil, water and wastewater to isolate bacteria, which are supposed to produce biosurfactant. Eighteen morphologically distinctive colonies of bacteria were isolated and purified. The isolation step shows that the most isolates obtained from wastewater treatment station. This may be returned to adaptation properties of microorganisms (Darwesh et al., 2019a). For more confirmation, one loop from the purified bacteria was spread on the surface of agar mMSM plates and incubated at 35 °C for 7 days. After incubation, the isolates from one to eighteen showed heavily growth except isolates no. 10 and 11, they have weakly growth. The nature of isolation is important to find adopted microbial community (Darwesh et al., 2020a). Thus, the successful isolation technology of biosurfactant-producing bacteria may signify the facility of microbes to survive in hydrocarbon contaminated areas and their ability for producing of biosurfactants (Primeia et al., 2020). Also, these isolates can represent naturally happening stress-resistant bacteria, successfully surviving in heavily polluted regions and might be utilized as a productive tool for bioremediation in future (Shoeb et al., 2013).

**Figure 1:**
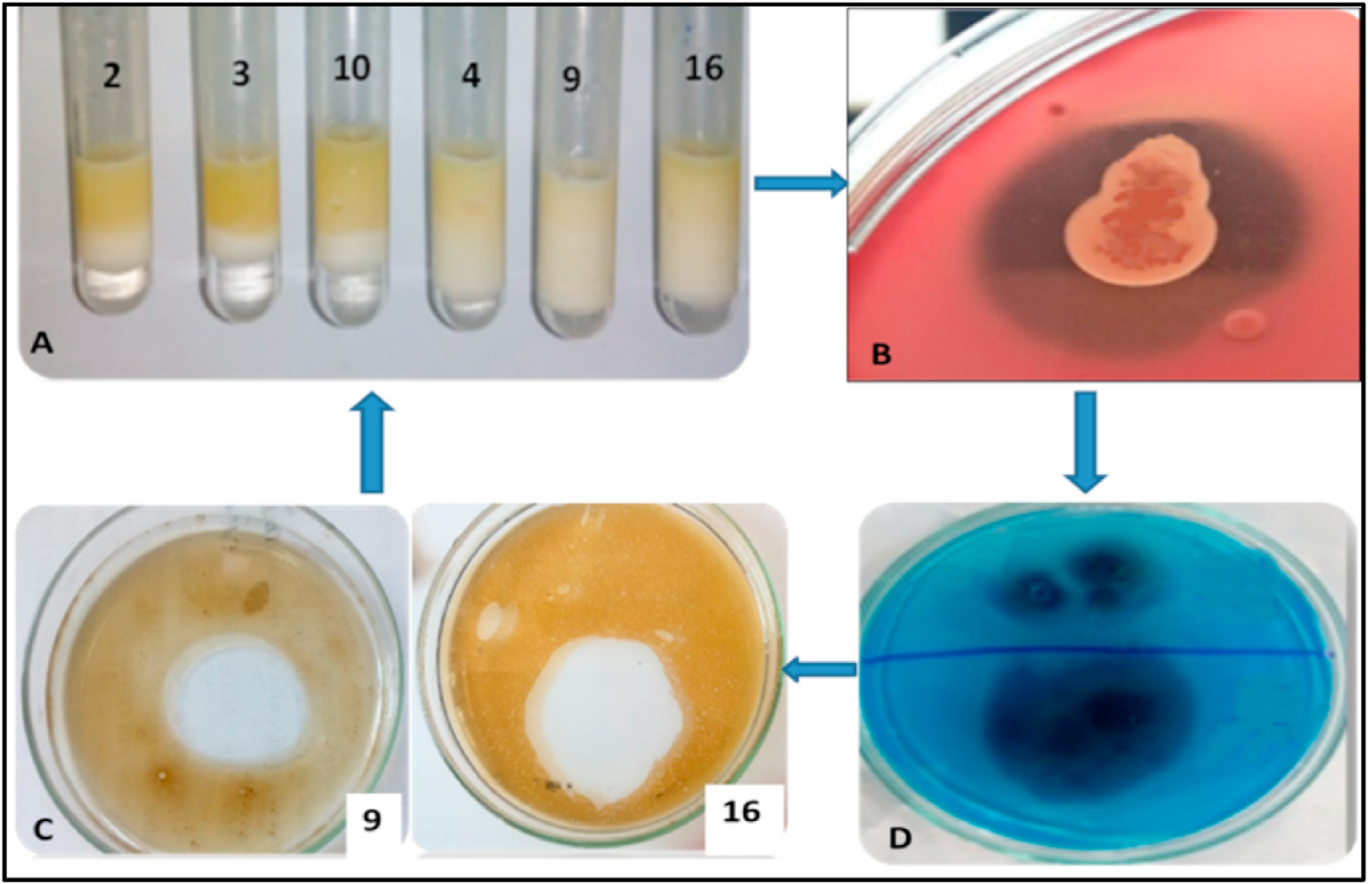
Screening methods for biosurfactant production by the obtained bacterial isolates; A, Emulsification test; B, Oil displacement test; C, blood hemolysis test; D, CTAP test.

Selection of the most powerful bacterial isolates was done based on their ability to produce high biosurfactant activity with different screening methods such as E_24_, ODT, HA, CTAB test and surface tension. The results of the screening methods were illustrated in Table 1. The most representative isolates for biosurfactant production were selected according to their effectiveness for more than one screening test. In case of E_24_ %, the supernatant of the obtained isolates after incubation in mMSM for 7 days was mixed with mineral oil and the emulsion layer height was measured as a determine factor for emulsification index (Figure 1A). The highest E_24_ % was recorded for isolates No. 1, 9 and 16 as 83, 80 and 80 %, respectively followed by the others (Table 1). In another way, the supernatant was added to the oil containing plates for testing the oil displacement properties (Figure 1C). The result illustrated that the highest oil displacement values were 1.2 and 2 cm oil clear zone for isolates 9 and 16, respectively. This technique used because it is rapid and easy to do where it's not need specific equipment and only required a small sample volume (Antoniou et al., 2015). In blood hemolysis test, the isolates were streaked on blood agar plate medium. The β-hemolytic activity (incomplete hemolysis) was noted in the bacterial isolate No. 9 (Figure 1B). The methylene blue agar plate supplemented with CTAB can be used to confirm found of anionic biosurfactant. Also, it is a semi-quantitative method for extracellular glycolipids detection (Xia et al., 2008). In the existing study, the pure culture of each isolate was streaked on MSM containing CTAB. The plate of bacterial isolate No. 16 appeared a dark blue halo around the colony indicating the anionic biosurfactant form as an insoluble complex with the cationic bromide salt, and the complex is revealed using methylene blue present in the agar (Figure 1D).

**Table 1:** Screening methods for biosurfactant production by the obtained bacterial isolates.

Isolates code	E_24_ (%)	ODT (cm)	HA (Haemolyses)	CTAP (zone)
1	83	0.3	N	NG
2	67	1	N	NG
3	67	1	N	NG
4	71	1.1	N	NG
5	78	0.5	N	NG
6	11	1	N	NG
7	67	0.8	N	NG
8	10	0.5	N	NG
9	80	1.2	++	NG
10	67	1	+	NG
11	17	0.3	N	NG
12	8	0.3	N	NG
13	17	0.6	+	NG
14	11	0.5	NG	NG
15	61	0.5	NG	NG
16	80	2	NG	+
17	11	0.3	N	NG
18	19	0.4	N	NG

∗NG: no growth, N: negative.

**Figure 2:**
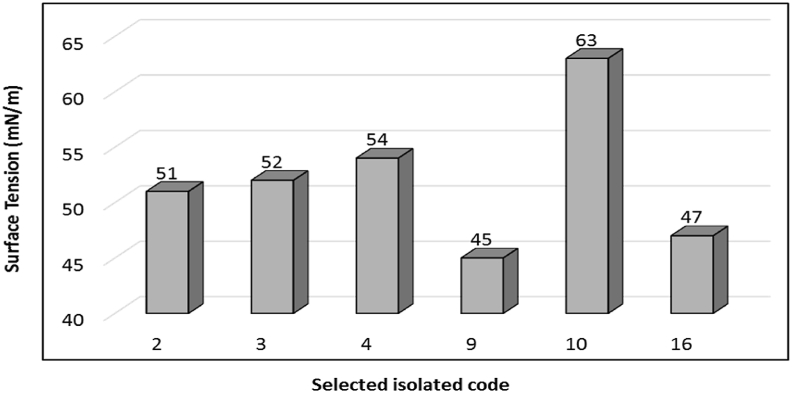
Surface tension determination for bacterial isolates producing biosurfactant.

The obtained results showed that the products of different isolates possess surfactant activity. The isolates recorded higher screening values in OST, E_24_%, HA and CTAP were selected for surface tension assay to confirm their biosurfactant production. The selected isolates were had code No. 2, 3, 4, 9, 10 and 16. The results illustrated in Figure 2 showed the most active isolates represented the highest surface tension were No. 9 and 16, which noted 45 and 47 mN/m, respectively. The surface tension reduction of the bacterial cultivation medium is considered the high surface tension activity (El-Sheshtawy et al., 2015). According to , a microorganism considered to be a promising as biosurfactant producer should be able to reduce the surface tension of the growth medium. The reduction of interfacial tension indicates the ability of microbial surfactants to remove oil from contaminated sites (Karlapudi et al., 2018). The interfacial tension is concentration dependent, as the aqueous solution concentration increased, the interfacial tension also reduced until the surfactant value is reached and remained constant there afterwards (Thavasi et al., 2011). The highest results recorded in oil spreading test, E_24_ and surface tension were isolate 9 and 16. For that, these isolates were subjected to identification section.

### 3.2 Identification of the potent bacterial isolates producing biosurfactant

**Figure 3:**
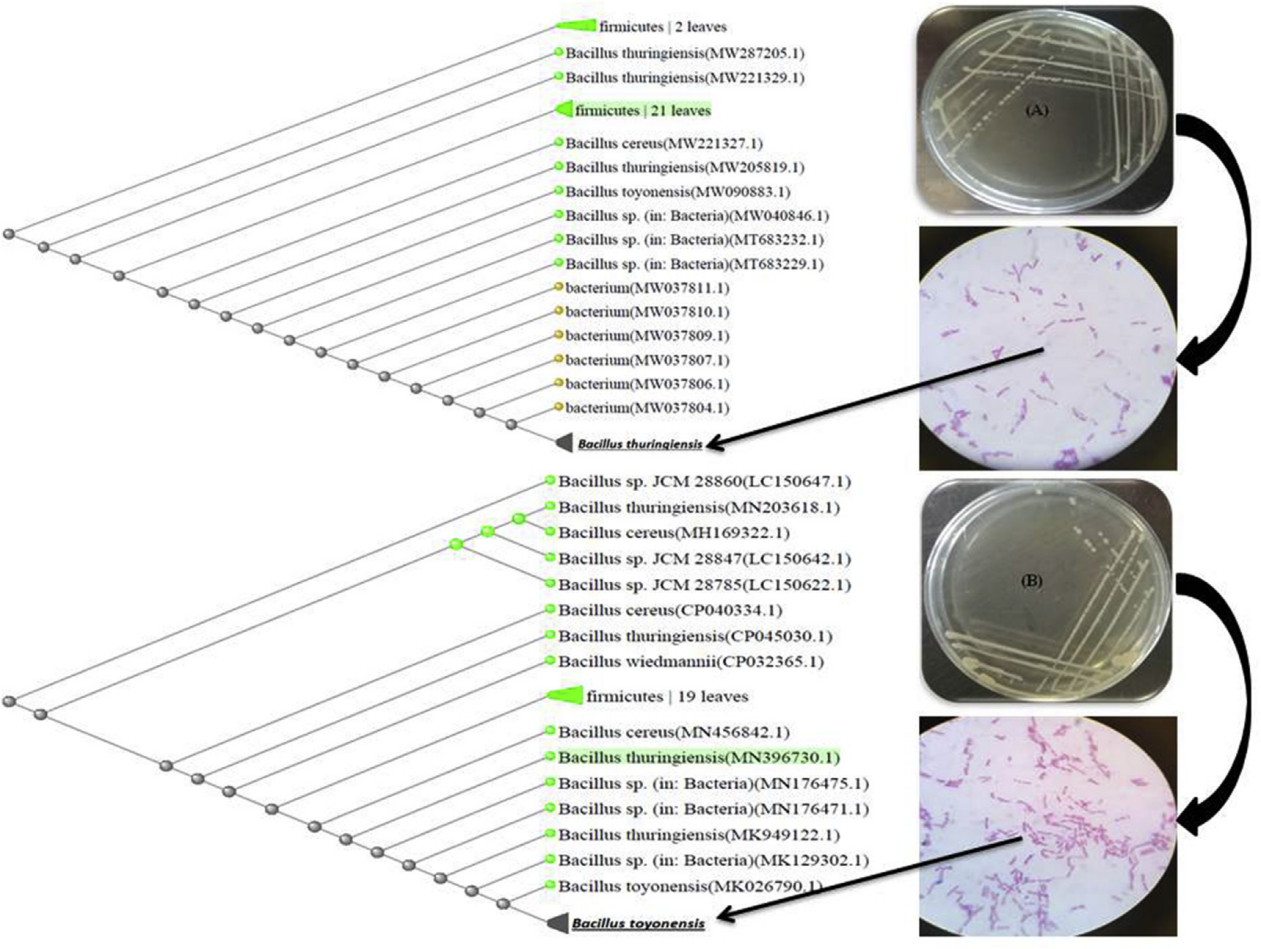
Microscopic examination and Neighbor joining phylogenetic tree constructed for the strains *Bacillus thuringiensis* isolate 9 (A) and *Bacillus toyonensis* isolate 16 (B) with other NCBI strains.

Two bacterial isolates, No. 9 and 16 were obtained as the most active biosurfactant producers. Therefore, it is important to identify these isolates. Colonies morphology of these isolates were examined using light microscope after gram staining. It was noted that the both isolates were rod shape and gram-positive. On the other way, they were spore former bacteria. Based on morphological and biochemical tests, both selected strains were found to be closely related to the species of Bacillus genus. In addition, molecular biology tools was performed for the both selected isolates to confirm identification. Genomic DNA was extracted and the 16s rRNA amplicon was obtained and then sequenced. The bacterial isolate No. 9 was identified as *Bacillus thuringiensis,* while the identification tools for isolate 16 led to identify it as *Bacillus toyonensis* (Figure 3). Also, the phylogenetic reconstruction was done using the neighbor joining algorithm as illustrated in Figure 3. At another work by Das et al. (2008), *Bacillus* sp. also has been stated as the main producer for biosurfactants like glycolipids, lipopeptides, halobacillin and surfactin.

### 3.3 Detection and quantification of biosurfactant

**Figure 4:**
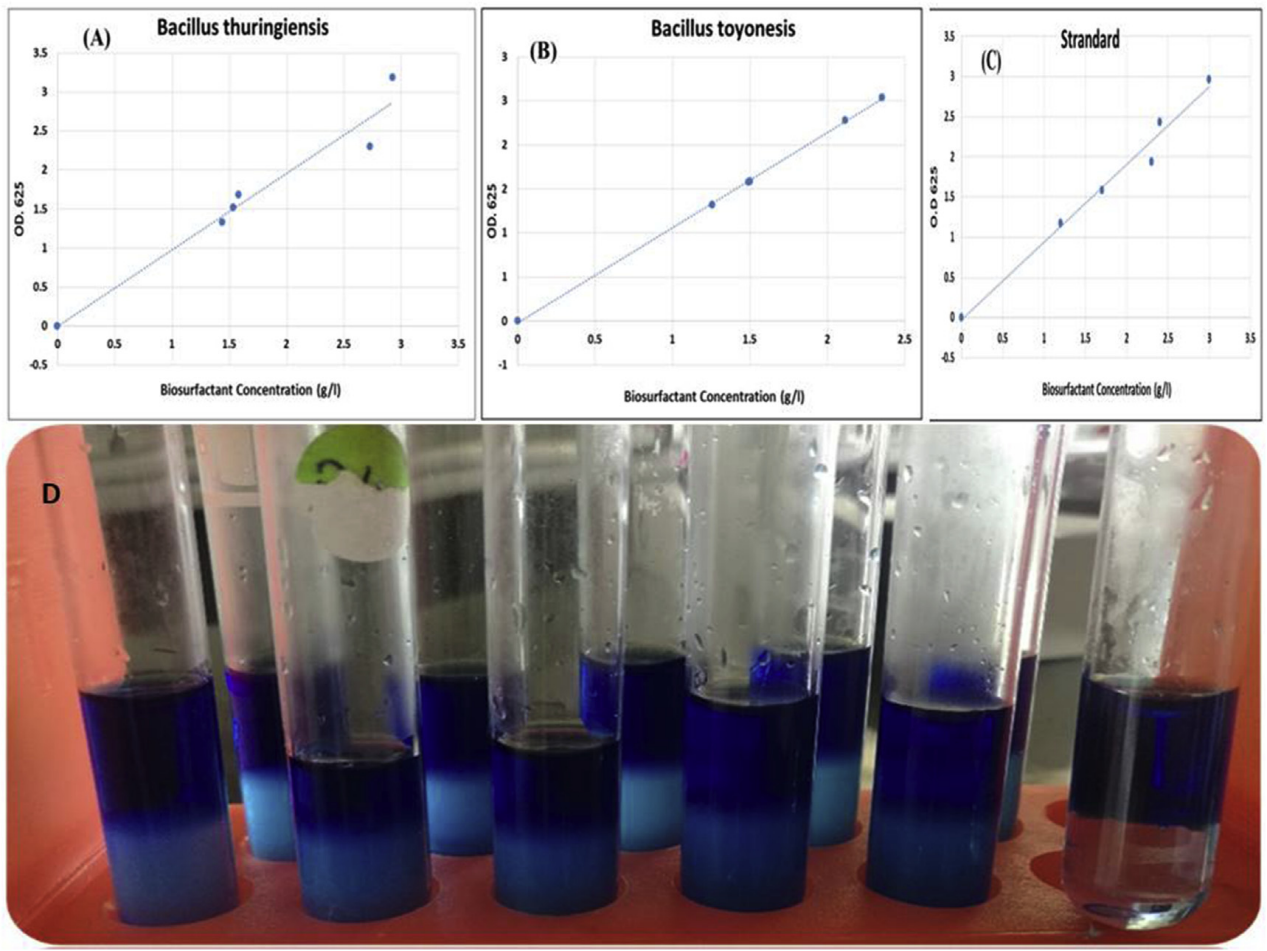
Standard curve of biosurfactant produced by *Bacillus thuringiensis* (A), *Bacillus toyonensis* (B) and SDS (C), in addition to the mixture of chloroform/biosurfactant (D).

Tulev and coworkers (2009) used the cell free filtrate (CFF) for extraction of methylene blue surfactant. The mixture of chloroform layer was represented in Figure 4, as well as the standard curve of standard surfactant (SDS) and the biosurfactant produced by the two strains after measuring at 625 nm toward chloroform as a reference. The biosurfactant concentration (g/l) was calculated using the slope of SDS standard curve and the following Eq. (1):

(1) Biosurfactant concentration (g/l) = (O.D – 0.0748)/0.8961

### 3.4 Optimization of incubation period to enhance biosurfactant productivity

**Figure 5:**
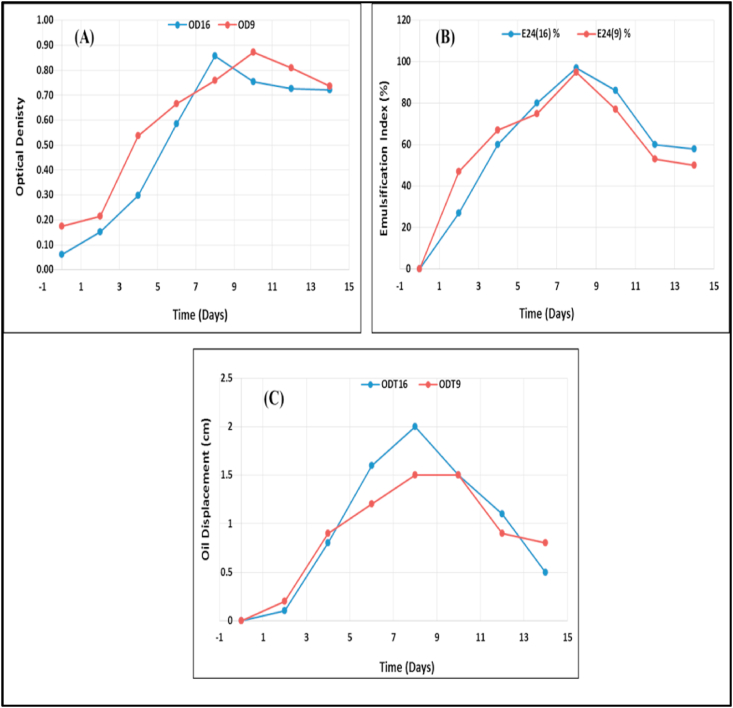
Effect of the incubation period on the biosurfactant productivity by *B. toyonensis* isolate 16 and *B. thuringiensis* isolate 9; where (A), Optical density; (B), Emulsification Index % and (C), Oil displacement (cm).

The study presented by Khopade et al. (2012) indicated that the biosurfactants are primary metabolites associated to cells biomass growth. Also, based on work of Amaral et al. (2006), the most biosurfactants were produced through cultures growth at stationary phase, while some species can produce them throughout exponential phase. On the other way, Lin and coworkers (2010) reported that some biosurfactants are produced as secondary metabolites. For that, the time of microbial growth is very important for production of their metabolites. In the existing study, the production of biosurfactant was measured at various incubation period times through 14 days. It is evaluated by oil displacement method, emulsification index and optical density for both strains, *Bacillus thuringiensis* and *Bacillus toyonensis* (Figure 5). The highest values for optical density, oil displacement and emulsification index for *Bacillus thuringiensis* were 0.856, 97 % and 2 cm, respectively after 8 days of incubation. While, the highest values for optical density, oil displacement and emulsification index for *Bacillus toyonensis* were 0.872, 95 % and 1.5 cm, respectively after 8 days of incubation (Figure 5). After the 10^th^ day, a gradual decrease of the biosurfactant activity was observed. The loss in activity was evidently determined after 2 weeks of incubation.

### 3.5 Optimization of biosurfactant production using Placket-Burman experimental design

**Figure 6:**
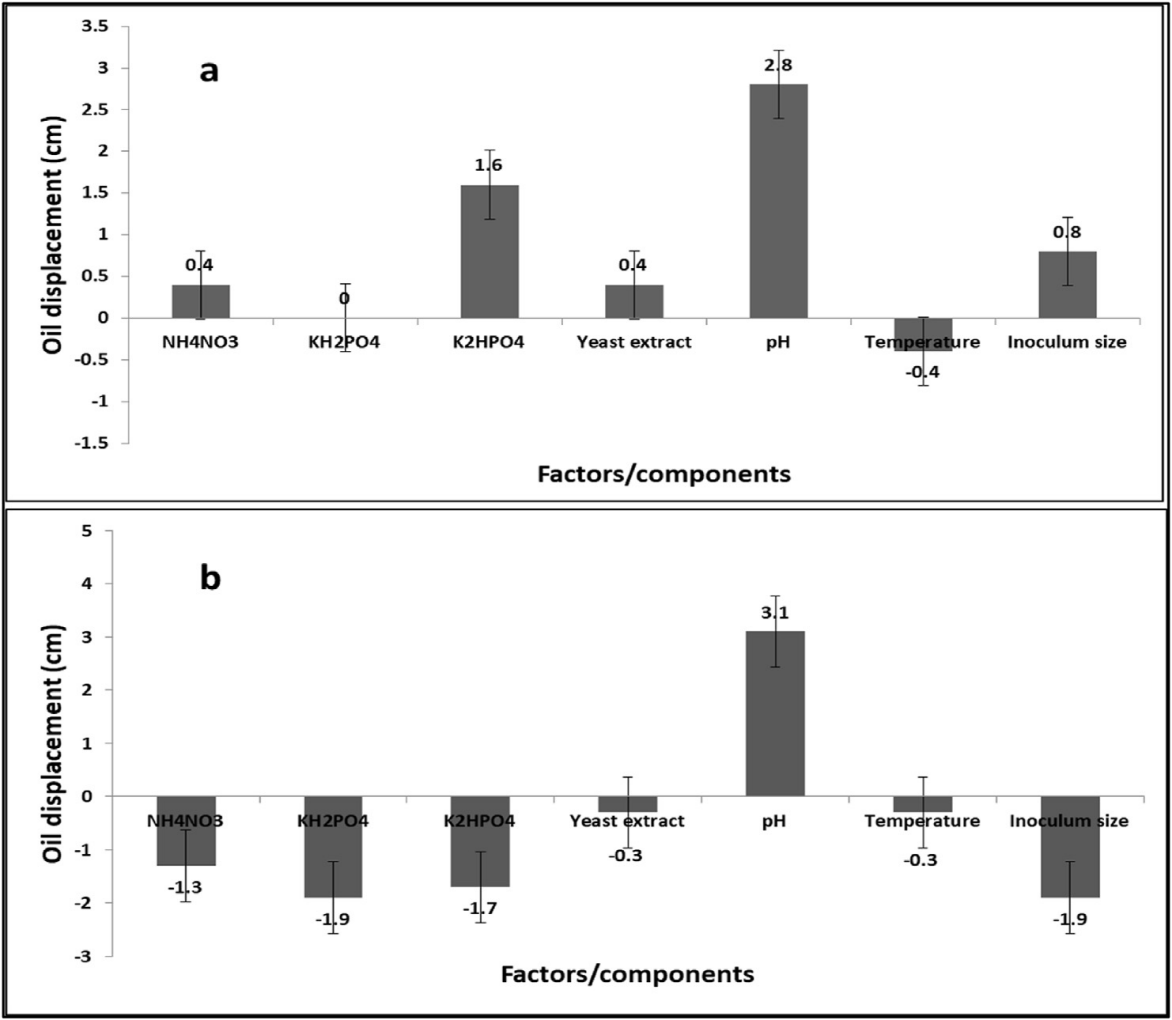
Statistical analysis of Placket-Burman design for maximum biosurfactant production by *B. thuringiensis* (a) and *B. toyonensis* (b) using oil displacement test.

Placket-Burman design is one of the popular statistical analysis method. This method applied here for detection the factors influenced biosurfactant production (El-Sersy, 2012). Many factors were studied to improve the productivity of biosurfactant. Nutritional conditions for microbial growing and metabolites manufacture differs amongst species. Hence, the design of specific supplies is very significant to increase the outputs. Enhancement of a process requires conditions optimization including numerous restrictions like media composition, pH and temperature degree (Korayem et al., 2015). Thus, Plackett–Burman design is frequently used for screening out significant factors and estimation their main effects. Both of oil displacement test and emulsification index used for recording the factors that influence biosurfactant production. The optimum conditions for maximum biosurfactant production for *B. thuringiensis* and *B. toyonensis* was recorded in Table 2 and the statistical analysis for the best result of oil displacement indicated that one factor was influenced biosurfactants production by *B. thuringiensis* (pH), where increasing the medium pH value lead to increase the production of biosurfactant (Figure 6a). In case of *Bacillus toyonensis*, the statistical analysis exposed that the pH was the most effective factor and the trend was moved like in another strain (Figure 6b). In this target, other researchers reported the same trend, with increasing the initial pH value of culture medium was produced increasing in biosurfactants productivity (Elshikh et al., 2016).

**Table 2:** The optimum conditions for maximum biosurfactant production by *B. thuringiensis* and *B. toyonensis* determined by oil displacement test.

Factor/components	Optimum condition
NH_4_NO_3_	6 g/L
KH_2_PO_4_	1.14 g/L
K_2_HPO_4_	1 g/L
Yeast extract	1 g/L
pH	9
Temperature	25
Inoculum size	0.5 mL

### 3.6 Production and characterization of the active purified biosurfactant agents

#### 3.6.1 GC-MS profile of biosurfactant extractable fraction from *Bacillus thuringiensis*

**Figure 7:**
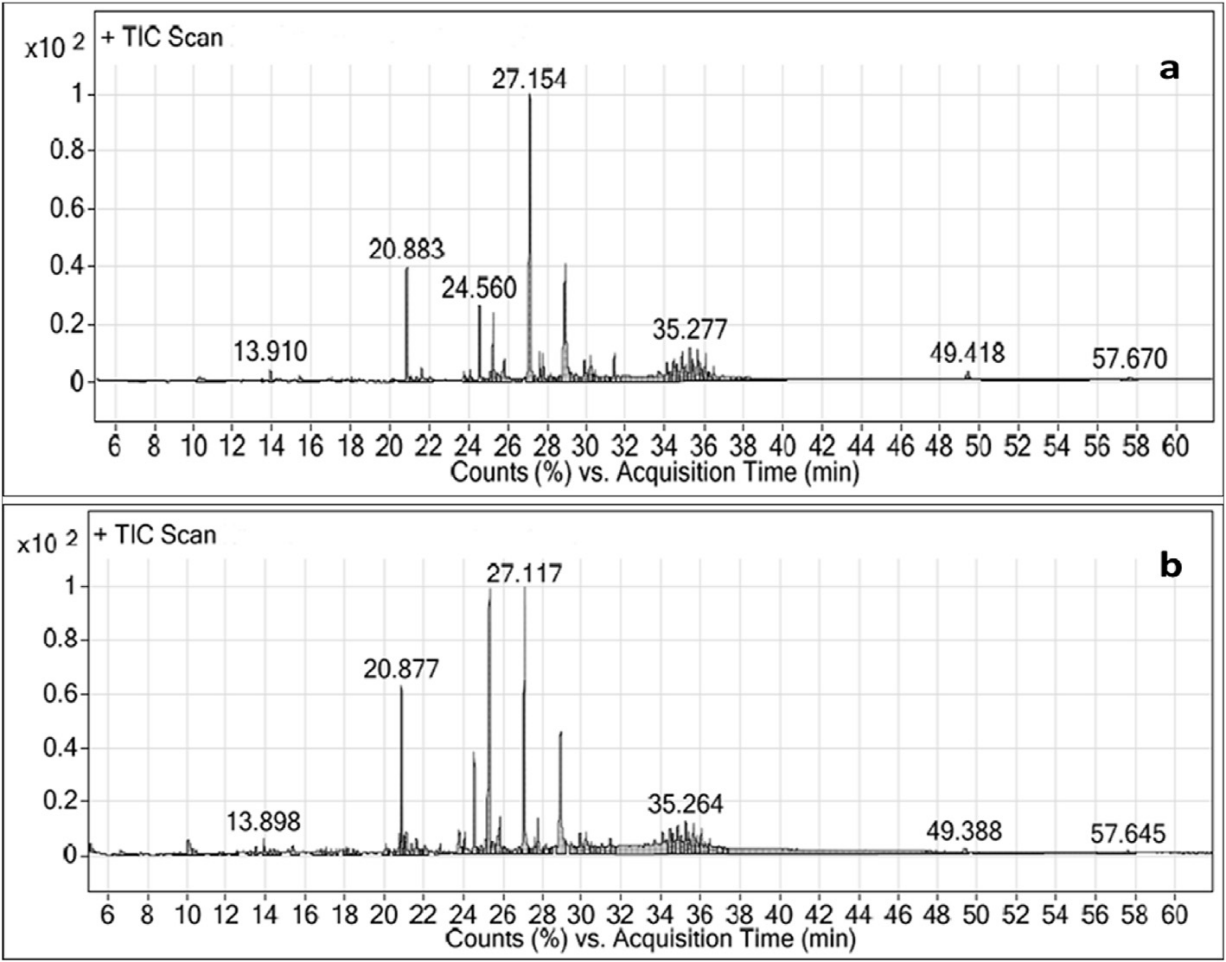
GC-MS profile for the biosurfactants produced by *B. thuringiensis* (a) and *B. toyonensis* (b).

The biosurfactant agents were produced under optimized conditions and the extraction technology was carried out based on combination between acid precipitation and solvent extraction (Vater et al., 2002). The obtained extracted samples was analyzed by GC-MS technique for identification of the produced compounds. From the profile of GC-MS analyses, 32 compounds were detected at different retention times (RT) from the sample produced by *B. thuringiensis* (Figure 7a). While, 22 compounds were identified with the sample produced by *B. toyonensis* (Figure 7b). The highest peaks recorded for the samples B16 and B9 with their corresponding compounds were listed in supplemented Table (S4 and S5). Among the major highest peaks compounds, decanoic acid was detected at RT of 20.877 min for both *B. thuringiensis* and *B. toyonensis*. Decanoic acid also known as capric acid is an ionic surfactant fatty acid with short alkyl tail (CH_3_ (CH_2_)_8_COOH).

Another major compounds recorded in this profile were oleamide which recorded at RT of 28.944 min in the sample produced by *B. thuringiensis* and oleic acid methyl ester which recorded at RT of 30.204 min for *B. toyonensis*. Oleamide is a non-ionic and nontoxic surfactant published by Abdel-Mawgoud et al. (2010) as white powder-like or flake-like with formula of C_18_H_35_NO. Also, it is inexplicable in water and organic solvents soluble and it has various kinds of properties including anti-adhesive, slipping, leveling, waterproof, moisture-proof, anti-settling, anti-fouling, anti-static electricity and dispersion. Oleic acid is a nonionic surfactant with formula of CH_3_(CH_2_)_7_CH = CH(CH_2_)_7_COOH. It is a fatty acid with odorless, colorless properties, although commercial samples may be yellowish.

#### 3.6.2 FTIR spectrum characterization

**Figure 8:**
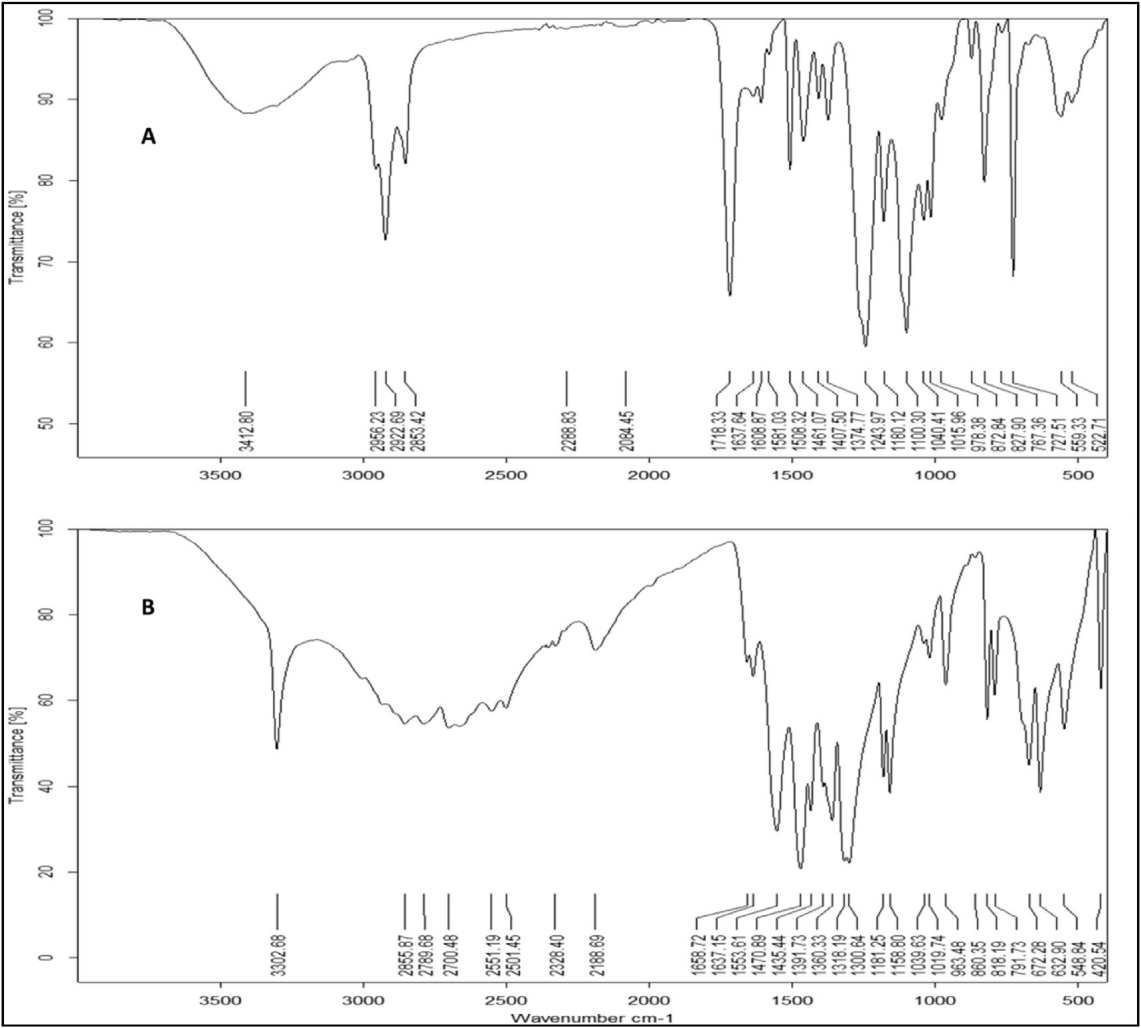
FTIR analyses of biosurfactant produced by *B. thuringiensis* (A) and *B. toyonensis* (B).

FTIR analysis was carried out for the both samples (produced by *B. thuringiensis* and *B. toyonensis* (Figure 8). Many bands were observed at different wavenumbers. For decanoic acid, the COOH group was detected as the carbonyl stretch (C=O) appeared between 1760-1690 cm^−1^, the C–O stretch appeared in the region of 1320–1210 cm^−1^, the O–H band was detected in the region of 1440–1395 cm^−1^ and 950-910 cm^−1^ and C–H bending appeared at 2800-3100 cm^−1^ (Liu et al., 2015). For oleamide, the amide group (C=O) occurs at around 1680–1630 cm^−1^, however, N–H group in primary amides (-NH_2_) stretches two bands neighboring 3350 and 3180 cm^−1^. The secondary amides have one band at 3300 cm^−1^, N–H bending occurs around 1640–1550 cm^−1^ for both primary and secondary amides. For oleic acid esters, C–O esters carboxylic acids displayed at 1300–1000, C=O stretch appeared in the range of 1750–1735 cm^−1^ for normal aliphatic esters. In case of conjugation between C=O and phenyl groups; 1740–1715 cm^−1^ for C–O and 1600–1450 cm^−1^ for ring were noted also (Figure 8). Carbonyl stretch, C=O of carboxylic acid appeared as an intense band from 1760 to 1690 cm^−1^, the C–O stretch appeared in the region of 1320–1210 cm^−1^, the O–H band was noted at 1440-1395 cm^−1^ and 950-910 cm^−1^ and C–H bending bands at 2800-3100 cm^−1^. Scissoring vibration of CH_2_ group adjoining carboxyl ester was also observed at 1351 cm^−1^ (Singh and Tiwary, 2016). The occurrence of aliphatic chains was established by observation of peaks in the region of 2850–2950 cm^−1^ due to find the –C–H stretching in the form of CH_3_ and CH_2_ groups in alkyl chains. The distortion ambiances from 1411 to 1270 cm^−1^ reflect aliphatic chains (–CH_3_, –CH_2_–) of the portion. This characteristically designated the presence of fatty acid as lipopeptide. A C–H stretching bands of –CH_2_ and –CH_3_ groups were observed in the region of 3000–2700 cm^−1^. The deformation vibrations at 1467 and 1379 cm^−1^ also confirmed the presence of alkyl groups (El-Sheshtawy et al., 2015). In both ranges, it is likely to observe bands for peptides characterization (wavelength of 3430 for NH, wavelength of 1655 for CO and wavelength of 1534 for CN) and aliphatic chains (wavelength of 3000–2800), representing that this composite is a lipopeptide (Das et al., 2008).

### 3.7 Biosurfactant stability

**Figure 9:**
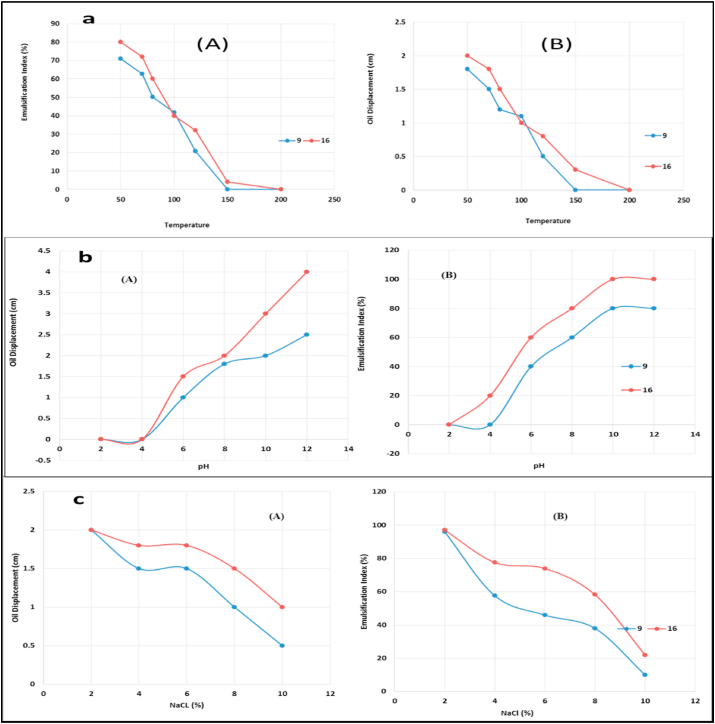
Stability of biosurfactant produced by *B. toyonensis* (9) and *B. thuringiensis* (16) toward heating (a), alkalinity (b) and salting (c) conditions evaluating using emulsification index (A) and oil displacement test (B).

To study the heat stability of biosurfactant produced by *B. thuringiensis* and *B. toyonensis*, the purified compound was heated for 15 min at different temperature degrees ranged between 50 and 200 °C using water bath. The results of E_24_ and oil displacement tests used to detect the thermal stability of biosurfactant were illustrated in Figure 9a. The data obtained and presented in this figure indicated that the biosurfactant from *B. thuringiensis* isolate 16 and *B. toyonensis* isolate 9 was stable at 50 to near 100 °C. Also, fasting decrease in E_24_ and oil displacement tests for both strains at the temperatures more than 100 °C. For its stability against pH value, the produced biosurfactant was evaluated its stability at different pH values ranged between 2 and 12. It was noticed that the produced biosurfactant agent from the both *Bacillus* strains was stable until pH 12 and reached the maximum value E24 = 80 % and 2 cm oil displacement for *B. toyonensis* biosurfactant and E24 = 100% and 4 cm oil displacement for *B. thuringiensis* (Figure 9b). The effect of NaCl concentrations on the extracted compounds was also examined. It was observed that the biosurfactant from both bacillus strains was stable until 8 % of NaCl concentration (Figure 9c). Based on the previous results, it can be decided the produced biosurfactant is halo-alkali-thermo agent. Fortunately, this biosurfactant stable at harsh conditions and can be used easy to remediate environment (Pacwa-Płociniczak et al., 2011).

### 3.8 Application of biosurfactant produced by *Bacillus* strains for remediation of wastewater contaminated with petroleum oil

Our previous published study stated that about 60–70 % of crude petroleum oils was found surrounding of oil tanks after predictable oil-recovery processes, also, distributed around oil stations and water/wastewater bodies and make harmful environmental problems (Barakat et al., 2017). It is important to remediate the contaminated sites using low-cost technology to introduce cleaner environment and available nontraditional water resource. Microbial or its related agents (like enzymes) degradation of oil contaminating sites is reported as green or cleaner low-cost technology (Darwesh et al., 2020c). The main limitation for large-scale application of oil-degrader microbes is to find surface-active compounds (biosurfactants) in surrounding contaminated area. Bioaugmentation of oil-degrading process is using various microorganisms community trapped in contaminated locations after enhancing by beneficial factors (Sayed et al., 2021). In current section of experiments, the microbial community of oil contaminating wastewater was enhancing using biosurfactant producing bacteria for degradation of oil. From the data tabulated in Table 3, we can stated that *B. thuringiensis* have best growth and biosurfactant production on the non-sterilized amended culture where E24 % and OST were 55 % and 1.1 cm, respectively. On the other hand, *B. toyonensis* have the best growth and biosurfactant productivity with sterilized amended culture where E24 % and OST were 65 % and 1.5 cm, respectively. In similar study on hydrocarbon degradation Nikolopoulou et al. (2013) reported that addition of rhamnolipids and inorganic nutrient in the presence of organic nutrients (uric acid and lecithin) was improved 3–3.5 times higher than the untreated samples. This is described the pivot role of biosurfactant to easy degradation of hydrocarbon, since it plays the cross-linker role with petroleum oil to increase its bioavailability resulting more degradability.

**Table 3:** Bioremediation of petroleum oil in contaminated wastewater using *B. thuringiensis* and *B. toyonensis* strains.

Culture Conditions	*Bacillus thuringiensis*		*Bacillus toyonensis*	
	E24 (%)	ODT (cm)	E24 (%)	ODT (cm)
Non-sterilized- non amended	15	0	25	0.1
Non-sterilized amended	**55**	**1.1**	25	0.1
Sterilized non amended	15	0	15	0
Sterilized amended	40	0.5	**65**	**1.5**

### 3.9 Antibacterial activity of the produced biosurfactant

Pathogenic microorganisms are reported the main dangerous factor in wastewater to limit use these wastewater after remediation (Abdelhameed et al., 2019). Thus, this part of result details the disinfection role of the produced biosurfactant as illustrated in Table 4 and (Fig. S3). Killing effect of materials is one of the major characteristics of biosurfactant application. The results of antibacterial efficiency of biosurfactant produced by *B. thuringiensis* and *B. toyonensis* against different types of bacteria showed positive indication against the tested strains by forming inhibition zone ranged between 8 and 15 mm without desk diameter. *Bacillus thuringiensis* showed activity against gram-positive bacteria (*St. pyogenes* and *B. cereus*) and gram negative (*Ps. aeruginosa* and *K. pneumonia*). Maximum inhibition zone was recorded by *B. thuringiensis* against *St. pyogenes, B. cereus* and *Ps. aeruginosa* ranged from 14 - 15 mm in compare with the different standards antibiotics. While, maximum inhibition formed by *Bacillus toyonensis* against the different kinds of gram-negative and gram-positive bacteria was recorded against *Ps. aeruginosa* as 13 mm.

**Table 4:** Antimicrobial activity of the biosurfactants produced by *Bacillus thuringiensis* and *Bacillus toyonensis* strains.

biosurfactant/Antibiotics	Inhibition zone (mm)					
	Gram positive isolates			Gram negative isolates		
	*B. cereus*	*S. aureus*	*St. pyogenes*	*Ps. aeruginosa*	*Acenetobacter*	*K. pneumonia*
Biosurfactant by *B. toyonensis*	0	8	11	13	0	12
Biosurfactant by *B. thuringiensis*	14	0	15	14	0	13
Amikacin	25	0	9	20	9	9
Amoxicillin/clavulanic acid	0	0	0	9	0	0
Ciprofloxacin	15	12	0	14	0	0
Piperacillin	22	0	0	20	8	0
Chloramphenicol	10	25	10	0	17	20

## 4 Conclusions

In conclusion, Based on the approved screening techniques out of 18 isolates, two bacterial isolates selected using the qualitative and quantitative tests as most active biosurfactant producers. They identified as novel *Bacillus thuringiensis* and *Bacillus toyonensis*, isolated from oil contaminated habitats, have the ability to produce stable biosurfactants, hydrocarbon degradation and antimicrobial activity. The produced biosurfactant has stability through 25 up to 120 °C, pH 9 up to 12 and NaCl concentration from 8 up to 10. The identified biosurfactant, Decanoic acid and oleamide can be used in remediation of oil contaminated sites as well as antimicrobial agent. Further studies can be done on the identified biosurfactants specially the highly recommended field of pest biological control and antifungal activity.

## Declarations

### Author contribution statement

Osama M. Darwesh, Mohamed S. Mahmoud, Kholoud M. Barakat, Ayman Abuellil & Maged E. Ahmad: Conceived and designed the experiments; Performed the experiments; Analyzed and interpreted the data; Contributed reagents, materials, analysis tools or data; Wrote the paper.

### Funding statement

This research did not receive any specific grant from funding agencies in the public, commercial, or not-for-profit sectors.

### Data availability statement

Data will be made available on request.

### Declaration of interests statement

The authors declare no conflict of interest.

### Additional information

No additional information is available for this paper.
