# Supplementary material for: Improving the bioremediation technology of contaminated wastewater using biosurfactants produced by novel bacillus isolates
Source: Heliyon. 2021 Dec 17;7(12):e08616. doi: 10.1016/j.heliyon.2021.e08616 (PMC8703238; doi:10.1016/j.heliyon.2021.e08616)
Supplement: supplementary data [file mmc1.docx]

**Supplementary data**


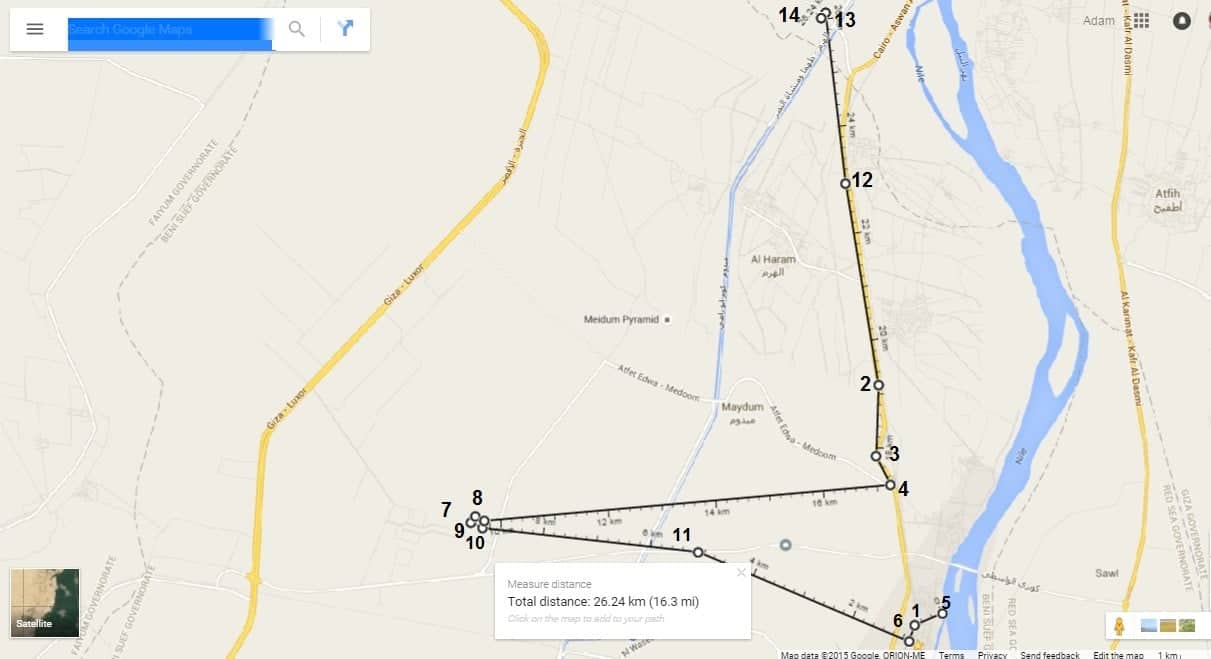


**Fig. S1.** Locations of oil-contaminated soil for isolation of biosurfactant producing bacteria

**Table S1.** Details of sampling areas and samples properties

| **Sample code** | **Address on Google map website** | **Nature of sample area** | **Address details** |
| --- | --- | --- | --- |
| **1** | 29°20'15.2"N 31°12'14.3"E | Real way station workshop | El-Wasta, Beni Suief, Egypt |
| **2** | 29°22'02.6"N 31°11'51.8"E | Gas Station | El-Wasta, Beni Suief, Egypt |
| **3** | 29°21'58.2"N 31°11'49.0"E | Cars workshop | El-Wasta, Beni Suief, Egypt |
| **4** | 29°21'38.8"N 31°12'00.6"E |  | El-Wasta, Beni Suief, Egypt |
| **5** | 29°20'16.8"N 31°12'34.6"E | Nile river beach | El-Wasta, Beni Suief, Egypt |
| **6** | 29°20'01.6"N 31°12'10.5"E | Gas Station | El-Wasta, Beni Suief, Egypt |
| **7,8,9,10** | 29°21'34.7"N 31°07'23.6"E | Wastewater treatment station | El-Wasta, Beni Suief, Egypt |
| **11** | 29°20'58.5"N 31°09'59.0"E | Gas Station | El-Wasta, Beni Suief, Egypt |
| **12** | 29°25'42.1"N 31°11'31.9"E | Cars workshop | El-Raqa Al Gharbeyah, Giza, Egypt |
| **13,14** | 29°26'25.3"N 31°10'42.9"E | Factory using oil | El- Raqa Al Gharbeyah, Giza, Egypt |

**Table S2.** Plackett-Burman design matrix for seven independent variables

| Run | NH_4_NO_3_ | KH_2_PO_4_ | K_2_HPO_4_ | Yeast Extract | pH | Temperature | Inoculum size (ml) |
| --- | --- | --- | --- | --- | --- | --- | --- |
| 1 | + | + | + | + | + | + | + |
| 2 | + | + | - | + | - | - | - |
| 3 | + | - | + | - | + | - | - |
| 4 | + | - | - | - | - | + | + |
| 5 | - | + | + | - | - | + | - |
| 6 | - | + | - | - | + | - | + |
| 7 | - | - | + | + | - | - | + |
| 8 | - | - | - | + | + | + | - |

**High (+) and Low (-) levels of different variables including medium components and culture conditions**

**Fig. S2.** Standard curve of standard biosurfactant (SDS g/l)

**Table S3**. Matrix of different MSM components concentrations and basic concentrations

| **Plackett-Burman** | | | | | | | |
| --- | --- | --- | --- | --- | --- | --- | --- |
| **MSM components** | **NH_4_NO_3_**  **(g/L)** | **KH_2_PO_4_ (g/L)** | **K_2_HPO_4_ (g/L)** | **YE**  **(g/L)** | **pH** | **Temp (C)** | **Inoculum size (ml)** |
| **Run** |  |  |  |  |  |  |  |
| **1** | 6 | 2.28 | 1 | 1 | 9 | 40 | 2 |
| **2** | 6 | 2.28 | 0.25 | 1 | 5 | 25 | 0.5 |
| **3** | 6 | 0.57 | 1 | 0.25 | 9 | 25 | 0.5 |
| **4** | 6 | 0.57 | 0.25 | 0.25 | 5 | 40 | 2 |
| **5** | 1.5 | 2.28 | 1 | 0.25 | 5 | 40 | 0.5 |
| **6** | 1.5 | 2.28 | 0.25 | 0.25 | 9 | 25 | 2 |
| **7** | 1.5 | 0.57 | 1 | 1 | 5 | 25 | 2 |
| **8** | 1.5 | 0.57 | 0.25 | 1 | 9 | 40 | 0.5 |
| **9 (control)** | 3 | 1.14 | 0.5 | 0.5 | 7 | 35 | 1 |

**Table S4.** GC-MS highest peaks for the sample produced by *Bacillus thuringiensis*

| Peak | Retention time (min) | Compounds | Area (%) |
| --- | --- | --- | --- |
| 1 | 20.877 | Tri decanoic acid, methyl ester | 23.93 |
| 2 | 24.553 | Methyl pentadecanoate | 17.27 |
| 3 | 25.267 | 1-Propene-1,2,3-tricarboxylic acid, tri butyl ester | 17.71 |
| 4 | 27.154 | Tri butyl acetyl citrate | 10.0 |
| 5 | 27.634 | Oxiraneoctanoic acid, 3-octyl-, methyl ester, cis- | 6.97 |
| 6 | 27.794 | Methyl 10-oxohexadecanoate | 8.3 |
| 7 | 28.944 | Oleamide | 59.05 |
| 8 | 34.877 | Phthalic acid, 5-methylhex-2-yl heptadecyl ester | 13.06 |

**Table S5.** GC-MS highest peaks for the sample produced by *Bacillus toyonensis*

| Peak | Retention time (min) | Compounds | Area (%) |
| --- | --- | --- | --- |
| 1 | 20.877 | Tridecanoic acid, methyl ester | 25.72 |
| 2 | 24.080 | Oleic acid | 9.05  5 |
| 3 | 24.553 | Penta decanoic acid, methyl ester | 17.34 |
| 4 | 25.322 | 1-Propene-1,2,3-tricarboxylic acid, tributyl ester | 59.24 |
| 5 | 25.845 | Butyl citrate | 14.08 |
| 6 | 27.124 | Tributyl acetyl citrate | 55.95 |
| 7 | 27.775 | Methyl 13,16-docosadienoate | 8.11 |
| 8 | 34.877 | Phthalic acid, bis(7-methyloctyl) ester | 10.76 |


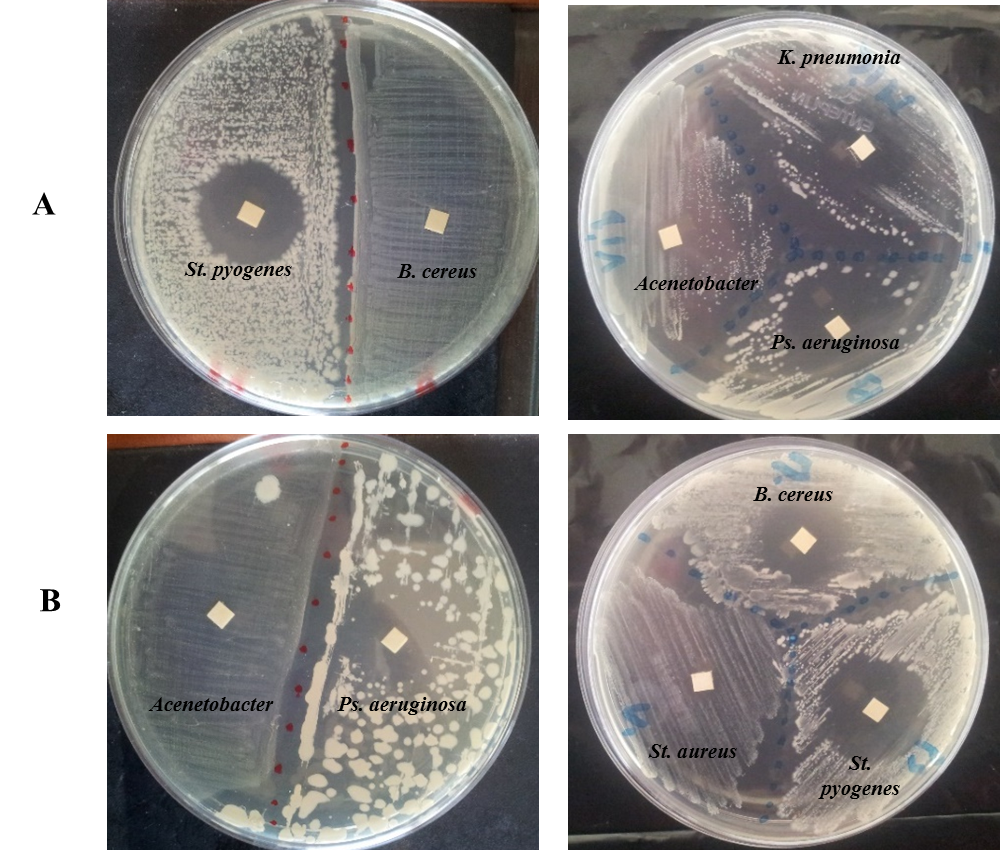


**Fig. S3.** Inhibition zone formed by biosurfactant extracted from *Bacillus toyonensis* (A) and *Bacillus thuringiensis* (B)
